# Cognitive Behavioral Therapy versus Short Psychodynamic Supportive Psychotherapy in the outpatient treatment of depression: a randomized controlled trial

**DOI:** 10.1186/1471-244X-7-58

**Published:** 2007-10-26

**Authors:** Ellen Driessen, Henricus L Van, Robert A Schoevers, Pim Cuijpers, Gerda van Aalst, Frank J Don, Mariëlle Hendriksen, Simone Kool, Pieter J Molenaar, Jaap Peen, Jack JM Dekker

**Affiliations:** 1 Depression Research Group, JellinekMentrum Mental Health Care, Overschiestraat 65, 1062 XD Amsterdam, The Netherlands; 2 Department of Clinical Psychology, VU University Amsterdam, Van der Boechorststraat 1, 1081 BT Amsterdam, The Netherlands

## Abstract

**Background:**

Previous research has shown that Short Psychodynamic Supportive Psychotherapy (SPSP) is an effective alternative to pharmacotherapy and combined treatment (SPSP and pharmacotherapy) in the treatment of depressed outpatients. The question remains, however, how Short Psychodynamic Supportive Psychotherapy compares with other established psychotherapy methods. The present study compares Short Psychodynamic Supportive Psychotherapy to the evidence-based Cognitive Behavioral Therapy in terms of acceptability, feasibility, and efficacy in the outpatient treatment of depression. Moreover, this study aims to identify clinical predictors that can distinguish patients who may benefit from either of these treatments in particular. This article outlines the study protocol. The results of the study, which is being currently carried out, will be presented as soon as they are available.

**Methods/Design:**

Adult outpatients with a main diagnosis of major depressive disorder or depressive disorder not otherwise specified according to DSM-IV criteria and mild to severe depressive symptoms (*Hamilton Depression Rating Scale *score ≥ 14) are randomly allocated to Short Psychodynamic Supportive Psychotherapy or Cognitive Behavioral Therapy. Both treatments are individual psychotherapies consisting of 16 sessions within 22 weeks. Assessments take place at baseline (week 0), during the treatment period (week 5 and 10) and at treatment termination (week 22). In addition, a follow-up assessment takes place one year after treatment start (week 52). Primary outcome measures are the number of patients refusing treatment (acceptability); the number of patients terminating treatment prematurely (feasibility); and the severity of depressive symptoms (efficacy) according to an independent rater, the clinician and the patient. Secondary outcome measures include general psychopathology, general psychotherapy outcome, pain, health-related quality of life, and cost-effectiveness. Clinical predictors of treatment outcome include demographic variables, psychiatric symptoms, cognitive and psychological patient characteristics and the quality of the therapeutic relationship.

**Discussion:**

This study evaluates Short Psychodynamic Supportive Psychotherapy as a treatment for depressed outpatients by comparing it to the established evidence-based treatment Cognitive Behavioral Therapy. Specific strengths of this study include its strong external validity and the clinical relevance of its research aims. Limitations of the study are discussed.

**Trial registration:**

Current Controlled Trails ISRCTN31263312

## Background

Depressive disorders constitute a major health problem in today's world. According to the World Health Organization, in the year 2000 depressive disorders were the leading cause of disability around the world and the fourth leading contributor to the global burden of disease. It is estimated that by the year 2020 depression will comprise the world's second largest disease burden, second only to ischemic heart disease [1]. Currently, more than 150 million people around the world are suffering from a depression [2]. Consequently, there is a high need for effective treatment.

The efficacy of existing psychotherapies for depressive disorders was recently reviewed by Roth & Fonagy [3]. They conclude that in general psychotherapy is an effective treatment of depression when compared to placebo. Cognitive Behavioral Therapy (CBT), Interpersonal Psychotherapy (IPT), Problem Solving Therapy (PST), couple therapy, bibliotherapy, and computer-aided therapy all have shown to be effective treatment methods, consistently efficacious in around 50–60% of cases. In contrast, there still is limited evidence base for brief dynamic therapy, although this form of treatment is widely applied in clinical practice. According to Roth & Fonagy, the results of the few available studies on brief dynamic therapy are flawed by methodological problems and a probable bias due to investigator alliance. The outcomes of good-quality trials, they conclude, suggest effectiveness equal to the psychotherapies mentioned above, but the conclusions that can be drawn about this treatment method are severely limited by the paucity of trials. This view is shared by the Cochrane reviewers of short-term psychodynamic therapies for common mental disorders [4]. They find modest to moderate gains of brief dynamic therapy for a variety of patients, but also conclude that these findings should be interpreted with caution because of limited data. Due to the scarcity of studies, the authors cannot draw any conclusions about the efficacy for depressed patients specifically. The present study aims at contributing to the gap in knowledge on this subject by comparing Short Psychodynamic Supportive Psychotherapy (SPSP) and Cognitive Behavioral Therapy in the treatment of depressed outpatients.

Short Psychodynamic Supportive Psychotherapy [5,6] was developed in the early 90's as a structured psychodynamically orientated treatment for depressed outpatients within JellinekMentrum Mental Health Care Amsterdam (JMHC). Since then the acceptability, feasibility, and efficacy of this treatment have been compared to pharmacotherapy and combined treatment (SPSP and pharmacotherapy) in four randomized clinical trials [7-10]. In these studies, treatment acceptability is conceptualized by the number of patients refusing treatment when allocated to it by study randomization. Feasibility is the number of patients who terminate treatment prematurely. Efficacy refers to the number of patients recovered from depressive symptoms according to an independent observer, the patient and the therapist.

De Maat et al. [11] performed a 'mega-analysis' on the data of the first three trials, in which the effects of SPSP, pharmacotherapy, and combined treatment were compared both in terms of symptom reduction and quality of life improvement. The results suggest that the combination of SPSP and pharmacotherapy is more efficacious than pharmacotherapy alone. Besides patients finding combined therapy more efficacious in reducing depressive symptoms, no difference in efficacy was found when comparing SPSP and combined therapy. SPSP and pharmacotherapy were found to be equally efficacious, except for some indications that patients and therapists favor SPSP with regard to symptom reduction. The results of the above-mentioned trials further indicate a better acceptability of SPSP compared to both pharmacotherapy and combined treatment [8,10]; fewer patients refuse SPSP because there is no medication involved. With regard to the feasibility, no differences were found [11].

In sum, previous research suggests that, while the combination of SPSP and pharmacotherapy seems to work better than pharmacotherapy alone, the superiority of combined treatment to SPSP is less obvious. In addition, SPSP and pharmacotherapy seem to be equally efficacious. Furthermore, the trial results provide support for the acceptability and feasibility of SPSP as an alternative treatment for depressed outpatients. Although combined treatment appears to be more efficacious than SPSP alone, this form of treatment is less well accepted by patients because of the required medication. Therefore SPSP might be a treatment of first choice for a great deal of depressed outpatients.

As mentioned earlier, so far SPSP has been compared to either pharmacotherapy or combined treatment. However, the question remains how SPSP compares with another established form of psychotherapy. Therefore the present study seeks to compare the acceptability, feasibility, and efficacy of SPSP to CBT, which is an evidence-based psychotherapy for the treatment of depressive disorders [3]. In addition, it is unclear whether there are specific groups of patients, who might benefit from one of these treatments in particular. This study aims to gain more insight into this issue as well.

### Research aims

The aim of this study is twofold. First, the research compares Short Psychodynamic Supportive Psychotherapy and Cognitive Behavioral Therapy in terms of acceptability, feasibility, and efficacy. Second, it seeks to identify clinical predictors that distinguish patients that can benefit from either of these treatments in particular. These clinical predictors include demographic variables, (comorbid) psychiatric symptoms, cognitive and psychological patient characteristics, and the quality of the therapeutic alliance.

### Hypotheses

Considering the first research aim, it is hypothesized that both treatments will be equally efficacious. This is based on Roth and Fonagy's [3] conclusions described above. In line with the earlier trials, it is further hypothesized that both psychotherapies will be equally acceptable and feasible to patients as well, since neither includes the use of medication.

With regard to the second aim, it is expected that a predictive relationship will be found between patient characteristics and the efficacy of one of the two treatments in particular. Because systematic research on other predictive patient characteristics is relatively scarce, only three hypotheses are formulated. Though based on a small dataset, Van et al. [12] found that a subgroup of patients with comorbid symptoms of anxiety benefited less from SPSP. It is hypothesized that CBT will be more effective for this group of patients, because CBT is generally considered to be the treatment of choice for anxiety disorders. In addition, it is hypothesized that patients with comorbid personality disorders may benefit more from SPSP. While these patients are usually regarded as difficult to treat, SPSP showed positive treatment effects when combined with pharmacotherapy in a subgroup of depressed patients with comorbid personality pathology [13]. Furthermore, it is thought that patients showing a higher degree of dysfunctional attitudes or cognitive reactivity to sad mood might respond better to CBT, because CBT specifically attends to these cognitive aspects.

## Methods/Design

### Design

This study is a randomized controlled trial comparing the acceptability, feasibility, and efficacy of Short Psychodynamic Supportive Psychotherapy (SPSP) and Cognitive Behavioral Therapy (CBT) in the treatment of depression. Participants are randomly allocated to either the SPSP or CBT treatment condition. Participants receive pharmacotherapy in addition to their psychotherapy if they show severe depressive symptoms at baseline assessment (*Hamilton Depression Rating Scale *[14,15]; HDRS score > 24). The main outcome measure is the number of patients with depressive symptoms in remission (HDRS score ≤ 8) at the termination of psychotherapy at week 22. In case of remaining depressive symptomatology at the termination of psychotherapy treatment (HDRS score ≥ 12 at week 22) patients will receive care as usual according to the JellinekMentrum Mental Health Care regular procedures. This usually consists of additional pharmacotherapy as described in more detail later.

### Participants/Setting

Participants are adult outpatients referred to one of three JellinekMentrum Mental Health Care (JMHC) clinics by their general practitioner on account of psychiatric complaints. These three mental health clinics are located in the city centre, the northern part, and the western part of Amsterdam (the Netherlands). It is therefore assumed that these clinics attend to a heterogeneous group of inhabitants.

Inclusion criteria are a main diagnosis of major depressive disorder or depressive disorder not otherwise specified (NOS), with or without a dysthymic disorder, according to DSM-IV criteria [16], mild to severe depressive symptoms (HDRS score ≥ 14 at base line), age between 18 and 65 years, and written informed consent.

Exclusion criteria are the presence of psychotic symptoms or a bipolar disorder, use of antidepressants, risk of suicide or loss of impulse control, substance misuse or abuse within the last six months (use of hard drugs, use of cannabis more than three times a week or alcohol above 21 units a week), use of antipsychotics or mood stabilizers, use of benzodiazepines (equivalent to more than 30 mg oxazepam per week), and use of medication that influences mental functions. Patients are also excluded from the trial if they are pregnant, not able to fill in the questionnaires because of language problems or physical difficulties, absent for more than three weeks during the treatment period or otherwise unable to complete the trial. Patients who were in contact with the same clinic within the last six months or participated in another depression research project within the last year cannot take part in the study either.

### Procedure

All patients referred to one of the three JMHC clinics are screened at intake by a psychiatrist and a psychologist for the presence of a depressive disorder and the absence of exclusion criteria. Eligible patients are invited for baseline assessment within one week after intake.

At the baseline assessment, inclusion and exclusion criteria are checked again, the research goals are explained, and information about participation in the research project is provided. Patients willing to participate sign an informed consent. Subsequently, the *Mini-International Neuropsychiatric Interview – Plus *(MINI-Plus) [17] and the *Hamilton Depression Rating Scale *(HDRS-17) [14,15] are conducted to confirm the presence of a depressive disorder and to determine the depression severity. Patients with a MINI-Plus diagnosis of depressive disorder and HDRS score of 14 or above are included in the study. They are randomly allocated to one of the psychotherapies, which are described in more detail in the next section, and are asked to fill in the questionnaires for the baseline measurement (see Table 1 for an overview).

**Table 1:** Instruments at different assessment moments

	**M1 Week 0**	**M2 Week 5**	**M3 Week 10**	**M4 Week 22**	**M5 Week 52**	**After each session**
**ASI**	X			X		
**BAI**	X		X	X	X	
**BSI**	X	X	X	X	X	
**CGI-S/I**					X	X
**DAS-A-NL**		X				
**DQ**	X					
**EQ-5D**	X		X	X	X	
**GAF**						X
**HAq**		X		X	X	
**HDRS**	X	X	X	X	X	
**IDS-SR**	X		X	X	X	
**LEIDS**	X			X		
**MAS**		X	X	X		
**MINI-Plus**	X					
**OQ-45**	X		X	X	X	
**PMS**		X				
**TFBq**				X		
**TiC-P**	X			X	X	
**VAS**	X	X	X	X		
**VKP**		X				

ASI: *Anxiety Sensitivity Index*, BAI: *Beck Anxiety Inventory*, BSI: *Brief Symptom Inventory*, CGI-S/I: *Clinical Global Impression Scale – Severity of Illness/Global Improvement*, DAS-A-NL: *Dysfunctional Attitude Scale*, DQ: *Demographic Questionnaire*, EQ-5D: *EuroQol*, GAF: *Global Assessment of Functioning Scale*, HAq: *Penn Helping Alliance Questionnaire Method*, HDRS-17: *Hamilton Depression Rating Scale*, IDS-SR: *Inventory of Depressive Symptomatology – Self-Report*, LEIDS: *Leiden Index of Depression Sensitivity*, MAS: *Medication Adherence Scheme*, MINI-Plus: *Mini-International Neuropsychiatric Interview – Plus*, OQ-45: *Outcome Questionnaire*, PMS: *Psychological Mindedness Scale*, TFBq: *Penn Therapist Facilitating Behaviors Questionnaire*, TiC-P: *Trimbos/iMTA Questionnaire for Costs associated with Psychiatric Illness*, VAS: *Visual Analogue Scales*, VKP: *Questionnaire on Personality Traits*

Within two weeks after the baseline measurement, psychotherapy starts. Participants with severe depressive symptoms (HDRS score > 24), who receive additional pharmacotherapy, have an appointment with a psychiatrist first. Assessments take place during the treatment period (week 5 and 10) and at treatment termination (week 22). In addition, participants are invited for a follow-up assessment one year after the start of treatment (week 52). At these assessments participants fill in questionnaires and the HDRS-17 is conducted by the independent assessor.

**Figure 1:**
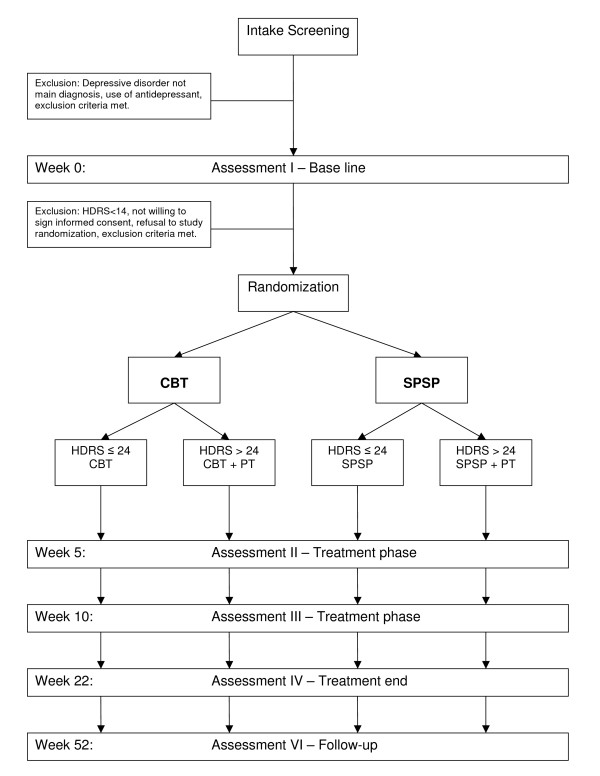
**Research procedure**. CBT: Cognitive Behavioral Therapy, HDRS: *Hamilton Depression Rating Scale*, PT: Pharmacotherapy, SPSP: Short Psychodynamic Supportive Psychotherapy

**Figure 2:**
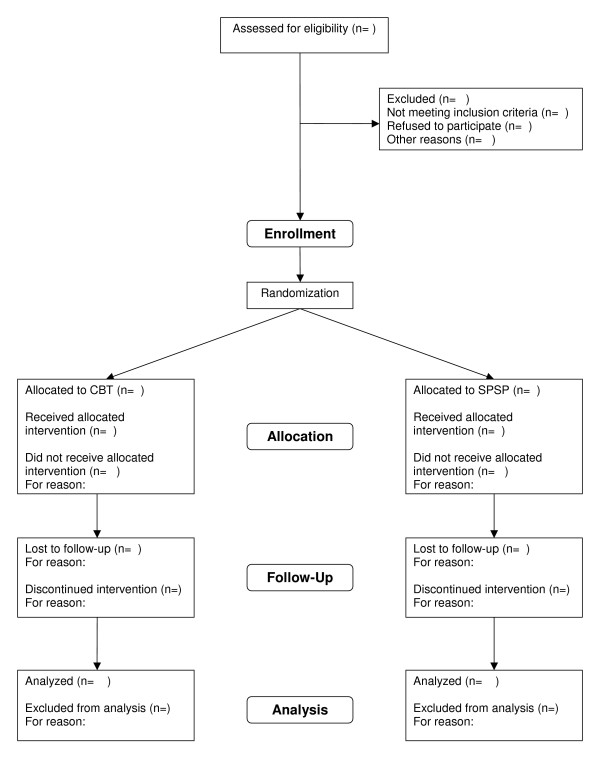
**Participant flowchart**. CBT: Cognitive Behavioral Therapy, SPSP: Short Psychodynamic Supportive Psychotherapy

Table 1 gives an overview of the interviews and questionnaires conducted at the different assessment times. Figure 1 represents the research procedure schematically. Figure 2 contains the expected participant flow.

### Interventions

Participants are randomly allocated to either Short Psychodynamic Supportive Psychotherapy (SPSP) or Cognitive Behavioral Therapy (CBT). Both are individual psychotherapies encompassing 16 sessions within 22 weeks. The first ten sessions take place weekly; the final six sessions two-weekly. The following paragraphs describe the theoretical background and treatment protocol of both therapies, the pharmacotherapy protocol, and the therapists.

#### Cognitive Behavioral Therapy

Cognitive Behavioral Therapy (CBT) was developed by Beck [18] and is founded in the cognitive theory, which states that information in the human brain is organized in certain patterns, or schemata, that contain general knowledge about the world and the person itself. These schemata are used to select, reduce, and interpret information. According to the cognitive theory, mental disorders are caused and maintained by dysfunctional thought schemata. Dysfunctional schemata express themselves in logical errors and dysfunctional automatic thoughts and give rise to all sorts of emotional and behavioral problems. Beck asserts that with depressive disorders, thinking in general is preoccupied with loss and hopelessness. The so-called depressive schemata are characterized by thoughts about one's own worthlessness and guilt, the world's cold-heartedness and injustice, and the future's desperateness. The cognitive part of CBT aims at locating and correcting the negative automatic thoughts and logical errors, and changing the schemata, thereby alleviating the depressive symptoms. Besides this cognitive element CBT contains a behavioral part, which is based on the notion that depression is partly caused or maintained by a lack of pleasant or satisfactory activities [19]. In CBT patients are therefore encouraged to identify activities that affect their mood positively and engage in these more often. CBT is further characterized by a limited time span, a structured approach, and the use of homework assignments.

The treatment protocol used in the present study is based on a CBT research protocol for the treatment of depressive and anxiety disorders [20], in which parts of the treatment protocol described by Boelens & Bloedjes [21] were adopted. The manual was rewritten solely for the purpose of treating depressive disorders. The main alteration for the current study is concerned with the emphasis on activation of the patient in the first treatment phase.

Cognitive Behavioral Therapy according to this protocol starts with one introductory session, in which acquaintance with the therapist is made, therapy conditions are explained and a treatment contract is signed by both the participant and the therapist. The CBT treatment itself consists of three phases. The first phase (sessions 2–4) focuses on the activation of the patient by means of planning and registering activities. In the second CBT phase (sessions 5–7) the cognitive model and the principles of cognitive therapy are explained. Patients keep a thought diary to identify automatic thoughts. These thoughts are challenged in the third phase (session 8–15), when they are tested on their validity and utility by logical reasoning together with the patient. Patients are encouraged to identify reasoning errors in their own thinking. In addition, a behavioral experiment is designed and conducted to test the identified automatic thoughts in real life. Depending on the patient's needs, sessions 13 to 15 can be spent on additional practice of basic challenging techniques, discussing complementary challenging techniques or paying further attention to the behavioral experiments. The final session (session 16) concludes treatment by evaluating the therapy itself and the therapeutic goals set at the start of treatment. When terminating the treatment, strategies of action in case of relapse are discussed as well.

#### Short Psychodynamic Supportive Psychotherapy

Short Psychodynamic Supportive Psychotherapy was developed as a treatment for depressed outpatients in the early '90s by de Jonghe [5,6]. SPSP is rooted in six psychoanalytical theories (described elsewhere [6]), which together assume six innate, basic, social needs: sexuality, aggression, the need to engage in relationships, and the needs to be protected, loved, and esteemed. If these needs are inadequately met in early infancy, they persist in adults as ongoing malignant aspects of the internal relationships, acting as moulds on new external and internal relationships. SPSP considers the gratification these needs particularly relevant in the treatment of depressed patients. The therapeutic action of SPSP consists in experiencing 'relational dissonance' or friction between two contradictory relationships simultaneously felt in the therapeutic situation. One is determined by moulds resulting from the past relationships, the other by the present relationship with the therapist, in which the patient will experience adequate gratification of his unmet early infantile need. The proper gratification of unmet developmental needs forms the psychoanalytical definition of 'support', which is considered the most important curative factor in SPSP [6,22].

In general, psychodynamically oriented psychotherapies can be placed on a supportive-expressive continuum. SPSP can be regarded as situated within the supportive half of this continuum. Psychodynamic therapies located on the expressive end emphasize the interpretation of transference. SPSP recognizes the existence of transference but does not interpret it.

Specific to SPSP is the distinction of nine different levels of discourse within the discussion of the problem area. The levels 1, 2 and 3 successively focus on the patient's physical and psychological symptoms and complaints, the influence of life circumstances on the depressive symptoms, and the influence of external interpersonal relationships on the depressive symptoms. At the fourth and fifth level the focus shifts to one or more relational patterns in the patient's life and the patient's attitude in life, respectively. The sixth level concerns how past relationships persist in the patient's current life, and the seventh level regards the intrapersonal relationship the patient maintains with himself or herself as a consequence of identification with these past relationships. At the eighth and ninth level the focus shifts to how the problems discussed at levels 4–7 manifest themselves in the relationship with the therapist. The levels of discourse can vary considerably during the course of treatment [6].

As mentioned above, support is regarded as the most important curative factor in SPSP. SPSP considers support advancing progression and maturing behavior as adequate, while regarding support advancing regression as inadequate. The therapist explicitly shows a supportive attitude by empathizing and being accepting, committed, active, flexible, clear, definite, patient, and persistent. In addition, the therapist systematically employs supportive techniques, such as reducing guilt, shame and isolation, clarifying, confronting, rationalizing, enhancing self-esteem, advising, and modeling.

Short Psychodynamic Supportive Psychotherapy as described by de Jonghe [23] constitutes the SPSP treatment protocol in the present study. According to this protocol SPSP consists of three treatment phases. In the starting phase the depressive complaints and their interpersonal context are attended to, psycho-education about depressive disorders is given, treatment aims are established, and a treatment proposal is made. The second phase is devoted to working on the treatment aims, which usually relate to one of four interpersonal problem areas; mourning, strife, role transformation or isolation. The problem area is discussed according to the different discourse levels. If possible a connection is made between the problems in this area and the internal relationships (level 3 and up). Patients are encouraged to experience their emotions and to reflect upon them. In addition patients are encouraged to change their behavior and cognitions, the consequences of which are discussed. The final phase deals with the treatment termination and possible related mourning. The treatment aims are evaluated, as well as the patient's perception of the treatment process. In addition, the patient's prognosis is considered and attention is given to confirmation of the patient's independence and handling of possible problems in the future.

#### Pharmacotherapy

Pharmacotherapy is given according to a fixed three-stage antidepressant protocol [24], which starts with the prescription of the antidepressant Venlafaxine XR 75 mg/day. This dose can be raised to reach the optimal medication effect with steps of 75 mg/day at each session until a maximum of 375 mg/day. In cases of severe side effects or inefficacy, Venlafaxine XR is decreased stepwise and Escitalopram is started at 10 mg/day without washout period. This dose can be raised once by another 10 mg/day to reach the optimal medication effect. In cases of severe side effects or inefficacy, Escitalopram is decreased stepwise and without washout period Nortriptyline 50 mg/day is started. This antidepressant is monitored by means of plasma levels (50–150 nanogram/l). In cases of severe side effects or inefficacy of Nortriptyline, the patient is considered as a study drop-out and treated outside the trial according to care as usual within JMHC.

Pharmacotherapy starts with a first visit to the pharmacotherapist, in which information about the pharmacology treatment is given, blood pressure is measured, and a treatment proposal is made. Then Venlafaxine XR is prescribed at a dose of 75 mg/day. For the first two weeks the patient visits the psychiatrist weekly. This frequency is reduced to two-weekly over the next six weeks and to once-monthly afterwards. Extra visits can take place when dose or medication are changed or whenever the psychiatrist considers this necessary. As the study period takes 22 weeks, further pharmacology steps will usually not take place during the trial phase.

#### Therapists

All pharmacotherapy therapists are either psychiatrists or resident psychiatrists. Psychotherapists in both the SPSP and CBT conditions are trained psychiatrists or psychologists, who all meet the educational criteria formulated by the JMHC Depression Research Group according to guidelines of the Dutch professional associations of psychotherapists. SPSP therapists and CBT therapists participate in one hour peer supervision groups on a two-weekly basis, in which audio taped sessions are discussed. Resident psychiatrists receive supervision weekly.

### Instruments

With regard to the instruments used in this study, primary outcome measures, secondary outcome measures, measures of clinical predictors and additional measures can be distinguished. The instruments include interviews, which are conducted by independent assessors, and self-report questionnaires, which are filled in by patients and therapists. Table 1 represents the interviews and questionnaires conducted at the different assessment moments.

#### Primary outcome measures

Primary outcome measures include the acceptability, feasibility and efficacy of treatment.

##### Treatment acceptability and feasibility

Acceptability of treatment is measured by the number of participants who refuse treatment when being randomly allocated to it. Feasibility is assessed by the number of patients terminating treatment prematurely.

##### Treatment efficacy

Treatment efficacy is measured by the decrease in depressive symptoms according to three different sources: an independent assessor, the patient, and the therapist.

Depression symptom severity according to the independent assessor is measured by means of the *Hamilton Depression Rating Scale *(HDRS-17) [14], which is a structured interview designed to quantify the severity of depressive symptoms in patients already diagnosed as suffering from a depressive disorder. Its 17 items cover different depressive symptoms, such as mood, sleep problems, lack of appetite, weight loss, suicide intentions, and feelings of guilt, which are rated on either a 0–2 or 0–4 scale. A psychometric review of the scale [25] concludes that: "the internal, interrater, and retest reliability for the overall Hamilton depression scale are mostly good" and "established criteria are met for convergent, discriminant, and predictive validity". The HDRS-17 is scored according to de Jonghe's scoring manual [26]. The independent assessors are research employees who engage in one hour peer supervision sessions two-weekly to promote interrater reliability. In these sessions audio taped interviews are discussed. If serious scoring problems arise, these are presented to the author of the Dutch scorings manual.

Depression severity from the patient's point of view is assessed by means of the *Inventory of Depressive Symptomatology, Self-Report *(IDS-SR) [27], which is a self-report questionnaire designed to measure specific signs and symptoms of depression in inpatients and outpatients. The scale consists of 28 items in five dimensions: vegetative symptoms, cognitive changes, mood disturbance, endogenous symptoms, and anxiety symptoms. Each of the items is rated from 0 to 3 and is equally weighted in the total score, which reflects the subjective severity of depressive symptoms. The IDS-SR has a good internal consistency, concurrent validity, and construct validity, which allows its use in research [27-29]. Furthermore, it has been proven to be sensitive to treatment effects in depressed outpatients [29].

As a measurement of the patient's functioning from the clinician's perspective the *Clinical Global Impression Scale *(CGI) [30] and the *Global Assessment of Functioning Scale *(GAF) [16] are used. The CGI is a widespread primary outcome measure in studies concerning the effectiveness of psychiatric treatments. It provides a summary of an individual's clinical functioning according to the therapist. The CGI consists of two ratings: *Severity of Illness *(CGI-S) at the moment of contact and *Global Improvement *(CGI-I) of the patient from the start of treatment. Both use a seven-step categorical scale "normal, not at all ill" (0) – "among the most extremely ill patients" (7) and "very much improved" (0) – "very much worse" (7), respectively. Therapists rate the CGI-I after each treatment session and the CGI-S after each session except for the first one.

The clinician's view of the patient's more general functioning is provided by means of the DSM-IV Axis V *Global Assessment of Functioning*. The GAF rates psychological, social, and professional functioning on a hypothetical continuum (0–100) from mental health to mental disorder. Therapists rate this instrument after the sessions 1, 5, 10, and 16, corresponding with the patient assessment moments.

#### Secondary outcome measures

Secondary outcome measures include general psychopathology, general psychotherapy outcome, pain, health-related quality of life, and cost-effectiveness.

##### General psychopathology

A shortened version of the *Symptom Checklist 90 *(SCL-90) [31], the *Brief Symptom Inventory *(BSI) [32] is applied as a measure of general psychopathology. The SCL-90 is the most frequently used instrument in mental health care to assess the nature and severity of psychopathology in adults. With its 53 items the BSI is remarkably shorter than the SCL-90, while conveying equal psychometric properties. A recent Dutch translation has been shown to have adequate reliability, good discriminant, and convergent validity and to be sensitive to treatment effect. The *Brief Symptom Inventory *assesses nine psychological symptom dimensions: somatization, obsessive-compulsive, interpersonal sensitivity, depression, anxiety, hostility, phobic anxiety, paranoid ideation and psychoticism. Each domain includes four to six items describing a complaint or symptom. Subjects are asked to rate the occurrence of this symptom in "the past week including today" on a five-point Likert scale from 0 (not at all) to 4 (extremely). In addition to the total scores on each dimension, the BSI yields three general indices to indicate the general level of psychological distress.

##### General psychotherapy outcome

General psychotherapy outcome is assessed using the *Outcome Questionnaire *(OQ-45) [33,34], which was developed to convey three domains central to mental health: symptom distress, interpersonal relations and social role functioning. The self-report questionnaire consists of 45 items to be rated on a five-point Likert scale ranging from 0 (never) to 4 (almost always). Reliability and validity of this instrument have been demonstrated [33,34].

##### Pain

To measure pain, numerical visual analogue scales (VAS) [35] are used. Following Fava et al. [36] scores are obtained on overall pain, headache, back pain, and shoulder pain. Respondents are asked to rate mean pain during the last week on a scale from 0 (no pain at all) to 10 (worst imaginable pain).

##### Health-related quality of life

The *EuroQol *(EQ-5D) [37] is a standardized, non-disease-specific, self-report instrument for describing health states. The EQ-5D questionnaire consists of 5 items covering the health dimensions: mobility, self care, usual activities, pain or discomfort, and anxiety or depression. The items follow the general form: 1 = no problems, 2 = some problems, 3 = extreme problems. In addition, a sixth item is included as a global perception of health status using a visual analogue scale ranging from 0 (worst imaginable health state) to 100 (best imaginable health state). Validity and reliability of the EQ-5D have been investigated and found to be acceptable [38-40].

##### Cost-effectiveness

Cost-effectiveness of both psychotherapies is evaluated using the *Trimbos/iMTA questionnaire for Costs associated with Psychiatric Illness *(TiC-P) [41]. The TiC-P is a self-report questionnaire to assess health care costs (part I) and costs resulting from production loss (part II) associated with psychiatric disorders. The 16 items of part I rate the number of contacts with different health care institutions within the last four weeks. Part II consists of the *Short Form Health and Labor Questionnaire *(SF-HLQ) [42] comprising three modules covering absence from paid employment, production loss without absence from paid employment, and impediments to paid or unpaid employment.

#### Measurement of clinical predictors

Clinical predictors, that might distinguish patients who benefit from either SPSP or CBT in particular, include demographic variables, psychiatric symptoms, cognitive and psychological characteristics, and the quality of the therapeutic alliance.

##### Demographic variables

A self-designed demographic questionnaire (DQ) is used to collect participants' demographic information. This instrument consists of 13 questions concerning nationality, ethnic origin, marital status, living situation, religion, education, occupation, and income.

##### Psychiatric symptoms

Concerning the clinical predictors of psychiatric symptoms, a distinction is made between depressive symptoms, comorbid anxiety symptoms and comorbid personality pathology. Measurements of depressive complaints include the HDRS-17, IDS-SR, CGI-I, CGI-S, and GAF, which are described above.

The presence of anxiety symptoms is monitored by use of the *Beck Anxiety Inventory *(BAI) [43], which is a self-report inventory for measuring the severity of anxiety symptoms in psychiatric patients. It includes 21 symptoms of anxiety, the presence of each within the last week to be rated on a four-point scale from 0 (not at all) to 3 (severely, I could barely stand it). The BAI shows high internal consistency and test-retest reliability, and evidence for its convergent and discriminant validity has been found [43,44].

The presence of personality disorders is screened by the *Vragenlijst voor kenmerken van de Persoonlijkheid [Questionnaire on Personality Traits] *(VKP) [45], which is a self-report instrument for the assessment of personality disorders based on the official World Health Organization instrument for the diagnosis of DSM-III-R and ICD-10 personality disorders, *International Personality Disorder Examination *(IPDE). The VKP consists of 174 items divided over seven areas, for example, work, affect and behavior, which concern the past 5 years and are scored on a three-point scale: true (2), ? (1), and false (0). VKP scores are moderately stable over time [45]. Because of the 2.5 time overestimation of the prevalence of personality disorders compared to a semi-structured clinical interview (IPDE), the VKP cannot be used as a diagnostic tool, but it is considered suitable as a screening instrument for personality disorders [46].

##### Cognitive and psychological patient characteristics

The cognitive characteristics monitored as possible clinical predictors of treatment effect include anxiety sensitivity, dysfunctional attitudes, and cognitive reactivity to sad mood. In addition, psychological mindedness is assessed as a possible predictive psychological patient characteristic.

Anxiety sensitivity refers to a person's beliefs that anxiety experiences have negative somatic, psychological or social consequences. In the present study this concept is measured by means of the *Anxiety Sensitivity Index *(ASI) [47]. Its 16 items are scores on a scale from 1 (very little) to 5 (very much). The ASI has been shown to have an acceptable internal consistency, good test/retest reliability, and a high degree of inter-item relatedness, although its factor structure remains somewhat controversial [47-50]. Moreover, ASI scores have been proven to be stable over a 10 month time-frame within a group of psychiatric outpatients with mood and anxiety disorders [50], and sensitive to treatment with antidepressants in depressed outpatients [51].

The Dutch version of the *Dysfunctional Attitude Scale *(DAS-A-NL) [52] is used as a self-report measure for the presence and intensity of dysfunctional attitudes or depressive suppositions, which are thought to constitute a vulnerability for the development of a depressive disorder. Subjects rate 40 statements regarding the way they usually look upon matters on a seven-point scale from 1 (totally agree) to 7 (totally disagree). The total score relates to the severity of dysfunctional attitudes. Internal consistency of this instrument is high and validity satisfactory [52].

The *Leiden Index of Depression Sensitivity *(LEIDS) [53] is a self-report questionnaire that aims to measure cognitive reactivity to sad mood, that is the relative ease with which maladaptive cognitions or cognitive styles are triggered by mild mood fluctuations. Participants are instructed to imagine a sad mood defined as: "a score of 3 or 4 on a scale of 0–10" and subsequently asked to rate 34 statements regarding their thoughts and behavior during this sad mood on a scale from 0 (not at all) to 5 (totally). The LEIDS contains four subscales: negative self-evaluation, acceptance/coping, indifference, and harm avoidance, and has been found to have good psychometric properties [53].

The *Psychological Mindedness Scale *(PMS) [54] is used in this study to measure the concept of psychological mindedness; an ability to access one's own and other's feelings and use these for changing behavior. Psychological mindedness as measured by this scale is generally regarded as a measure of patients' suitability for dynamically orientated psychotherapy. The scale contains 45 self-report items scored on a 4-point scale ranging from 1 (strongly disagree) to 4 (strongly agree). The PMS has good internal consistency, and the total score has been found to be correlated with the number of psychotherapy sessions attended by psychiatric patients [54-56].

##### Therapeutic alliance

Therapeutic alliance, or helping alliance, from the patient's perspective is measured by means of the *Penn Helping Alliance Questionnaire Method *(HAq) [57,58], which assesses the extent to which the patient experiences the therapist and the therapy as helpful. Therapeutic alliance from the therapist's perspective is assessed by means of the *Penn Therapist Facilitating Behaviors Questionnaire *(TFBq), which measures the degree to which the therapist feels that he or she is helping the patient [58]. Both instruments are part of the *Penn Helping Alliance Scales*, which distinguish two types of helping alliance. Helping Alliance Type 1 refers to the patient's perceived helpfulness of the therapist, whereas Helping Alliance Type 2 is defined as the patient's collaboration or bonding with the therapist. The HAq and the TFBq are parallel self-report instruments including 11 items; 8 relating to Helping Alliance Type 1 and 3 relating to Type 2. Each item is rated on a 6-point scale from -3 (No, I strongly feel that it is not true) to 3 (Yes, I strongly feel that it is true). The total score equals the sum of the item ratings. The *Penn Helping Alliance Scales *show adequate overall reliability [59]. Therapeutic alliance, both in general [59] as well as measured by the HAq and the TFBq in the early phases of treatment [57], has been found to be correlated with psychotherapy outcome.

#### Additional measures

In addition to the primary and secondary outcome measures and the instruments aimed at identifying clinical predictors of treatment effect, two instruments are included in order to confirm the clinician's diagnosis of depressive disorder and to monitor medication adherence. Additional measures also include a number of instruments which are part of the standard monitoring procedure of JMHC. These instruments will be used in secondary analyses.

##### Diagnosis

In addition to the clinician's diagnosis the *Mini-International Neuropsychiatric Interview – Plus *(MINI-Plus) *Sections A: Major Depressive Episode *and *B: Dysthymic Disorder *[17] is conducted. The MINI-Plus is a clinician-rated structured interview designed to diagnose psychiatric disorders according to DSM-IV and ICD-10 criteria. Both sections focus on current symptoms, and positive answering to two screening questions is requisite to proceed to the remaining questions concerning the complaints in more detail. The MINI-Plus diagnostic interview is in widespread use, because of its reliability and validity in diagnosing DSM-IV and ICD-10 psychiatric disorders in a short time [17].

##### Medication adherence

Medication adherence is monitored at every assessment moment for participants using antidepressants, by means of the *Medication Adherence Scheme *(MAS). After having explained that forgetting or not taking medication is common and that the information given will not reach the pharmacotherapist, the independent assessor questions the participant about the prescribed dose of antidepressant and the amount not taken accidentally or on purpose. Results are registered in the scheme, from which the percentage of medication taken is calculated by dividing the amount of medication taken by the total amount of medication prescribed.

##### JMHC standard monitor instruments

The JMHC employs a routine monitor procedure for all its patients in order to monitor treatment effects, with assessment moments coinciding with the assessment moments of the present study (week 0, week 22, and week 52). Data from this standard procedure will be available to perform secondary analyses on outcome measurement and clinical predictors. The standard monitoring procedure covers the areas of treatment requests, personality traits, coping behavior, mastery, acculturation/discrimination, life events and general satisfaction with mental health care. The instruments include the *Patient Request Form *(PBV) [60], *Dutch MMPI short version *(NVM) [61], the *NEO Five Factor Inventory *subscales *Neuroticism *and *Extraversion *(NEO-FFI) [62], *Utrecht Coping Questionnaire *subscales *Active Coping *and *Avoidant Coping *(UCL) [63], the abbreviated Dutch version of the Personal Mastery Scale (PMS-D) [64], the *Lowlands Acculturation Scale *(LAS) [65], the *List of Threatening Experiences *(LTE) [66], and the *Mental Health Care Thermometer *(MHCT) [67].

### Randomization

Randomization is stratified into two gender groups (male/female) and two age groups (<32.5/>32.5). The independent research assessors perform the enrollment and randomization of the patients. Randomization is executed according to a list, the allocation sequence of which was computer-generated by one of the researchers (JP).

### Power

To detect a 15% difference in effectiveness between the SPSP and CBT conditions with α = .05 and β = .80, 150 participants in each treatment condition are needed.

### Analyses

Group differences will be investigated by chi-square tests, an(c)ova, man(c)ova, and survival analyses. The analyses will be performed according to the principles of *intention to treat *and *per protocol *design. In addition, *completer's analyses *will be conducted. Missing data will be replaced by either the data of the last available measurement using the principle of *last observation carried forward*, or by using more sophisticated imputation techniques, for instance multiple imputation or regression imputation.

The costs-utility ratio will be graphically represented in a 95%-confidence ellipse.

Sensitivity analyses will be carried out to determine the robustness of the results under different conditions, varying the primary variables. Patient variables predicting treatment outcome will be identified using multiple regression analysis and logistic regression analysis.

### Ethical principles

This study complies with the principles of the Helsinki Declaration [68]. The design and execution of this study were approved by the Dutch Union of Medical-Ethic Trail Committees for mental health organizations.

Participation is voluntary and patients are informed that they can withdraw their consent to participate at any time, without any consequences for their further treatment. Participants are informed about the research aims and informed consent is obtained prior to the assessment of patient eligibility.

Confidential information and patient names are treated according to the medical confidentiality rules. Patient data and patient details are stored in different files. All study related documents and data are kept on the protected central server of JellinekMentrum Mental Health Care, with access limited to members of the research team.

## Discussion

This paper describes the study protocol of a randomized controlled trial concerning Cognitive Behavioral Therapy versus Short Psychodynamic Supportive Psychotherapy in the outpatient treatment of depressive disorders. The aims of this study are to compare both treatments in terms of acceptability, feasibility and efficacy, and to identify clinical predictors that distinguish patients that may benefit from either of these treatments in particular.

The study design described above has specific strengths and limitations. First of all, a strength of this study is that both research aims relate to important matters in the treatment of depressed outpatients. Concerning the first research aim, there is a general paucity of studies regarding the effectiveness of brief dynamic therapy in the treatment of depression, limiting the evidence base of this treatment method. More specifically, this study compares two psychotherapy treatments, which have never been directly compared before. To date, Short Psychodynamic Supportive Psychotherapy has only been directly compared to either pharmacotherapy or combined treatment (SPSP and pharmacotherapy). In both respects, this study relates to research questions unanswered so far. With regard to the second aim, this study does not only assess the effectiveness of the treatments, but it also hopes to gain more insight into the question as to which type of treatment will be most suitable for which type of patient. Therefore the results of this study can be used in clinical practice to improve the treatment allocation of patients.

Secondly, a strength of this trial is its strong external validity. Randomized clinical trials are often criticized for their artificial treatment conditions, which make generalization of the results difficult. In this study the external validity is specifically attended to, resulting in outcomes that might be directly relevant for clinical practice. The external validity is supported by the study's design, the outcome measures, the participant selection, and its multi centered character. With regard to the design, this study uses a *comparative strategy*, which directly compares two fully realized clinically representative treatment packages. Participants receive psychotherapy treatment in a regular outpatient clinic in the same way they would have received it had they not been involved in the research project. Therefore, the results of this study will directly apply to the treatment in practice. Furthermore, the strong external validity is reflected in the outcome measures. The treatments are not only evaluated on efficacy, but acceptability and feasibility are also assessed. Both the latter are important issues in the effectiveness of treatment in clinical practice. In addition, the primary outcome measure, reduction of depressive symptoms, is measured from three different perspectives. Besides an independent researcher (HDRS-17), the therapist (CGI-I/S and GAF) and the patient (IDS-SR) rate the improvement, thereby creating a full picture of the treatment effects. Finally, external validity is supported by this trial's wide participant selection and multi centered character. Participants are not specifically selected on their suitability for psychotherapy. Therefore, they represent a general population of psychiatric patients rather than a selected group of patients. By including patients from three different clinics in different parts of the city, a heterogeneous population is studied, contributing to the generalization of this study's results.

A third strength of the present study consists in its regulations to increase internal validity. Internal validity is promoted by specific attention given to therapists' adherence to the treatment protocol, the interrater reliability of the main primary outcome measure, and the use of reliable and valid instruments. Concerning the adherence to the treatment protocol, internal validity is seen to by the arrangements of fortnightly supervision groups for psychotherapists in which audio taped sessions are discussed. In addition, the interrater reliability of *Hamilton Depression Rating Scale *assessors are also specifically attended to by two-weekly peer supervision groups, with scoring problems being presented to the author of the Dutch scorings manual. With regard to the measurement of outcome and patients' characteristics, only widely used instruments with good psychometric properties are included in this study.

Regarding the limitations of this study three comments must be made. In the first place, as noted earlier, the study uses a *comparative strategy*, which directly compares two fully realized clinically representative treatment packages. Although this strategy is beneficial to the external validity, it is impossible to identify specific operative aspects within the treatment. Consequently, this study does not focus on these aspects. In the second place, a limitation of the current study is the absence of a control group or waiting list condition. Besides the ethical considerations of withholding patients from treatment for 22 weeks, it was practically very difficult to incorporate such a condition into this design. A third limitation is the fact that outcome assessors are not blinded for treatment conditions. Although blinding undoubtedly would have contributed to the internal validity of this study, it is by definition impossible to blind patients and therapists for psychotherapy treatment conditions. Because the independent research assessors work in small-scale clinics, it was impossible to prevent them from knowing the therapists' treatment conditions. Therefore, the independent research assessors could not be blinded. Nonetheless, statistical analyses will be performed blinded in order to minimize the information bias effects.

Depression constitutes a major health care problem in today's world. This study aims to contribute to the evidence-based treatment of this disorder by further investigating a potential promising form of psychotherapy. By expanding the knowledge about cognitive behavioral versus psychodynamic treatment of depressive disorder, and about which patients might benefit from one of these treatments in particular, a still existing gap in knowledge will hopefully be further filled.

## Competing interests

The authors declare that they have no competing interests.

## Authors' contributions

ED coordinates the data acquisition and wrote the manuscript.

FJD supervises Cognitive Behavioral Therapy.

GvA, RAS and HLV devised the pharmacotherapy protocol and supervise pharmacotherapy.

HLV, MH, and SK supervise Short Psychodynamic Supportive Psychotherapy.

JJMD leads the research project.

JJMD, HLV and RAS developed the study design and wrote the study proposal.

JP developed the statistical design and randomization procedure, manages the data flow and will perform the statistical analyses.

PC contributed to the development of the research aims and design.

PJM and FJD wrote the CBT protocol.

All authors provided comments, read and approved the final manuscript

## Pre-publication history

The pre-publication history for this paper can be accessed here:


